# Experimental and Numerical Investigations on Thermal-Hydraulic Performance of Three-Dimensional Overall Jagged Internal Finned Tubes

**DOI:** 10.3390/mi15040513

**Published:** 2024-04-11

**Authors:** Shufeng Huang, Mingjiang Deng, Zhixin Chen, Dayong Yang, Yingshuai Xu, Ning Lan

**Affiliations:** 1 School of Mechanical and Electronic Engineering, East China University of Technology, Nanchang 330013, Chinaxuyingshuai@ecut.edu.cn (Y.X.); ln66645@163.com (N.L.); 2 School of Mechanical and Automotive Engineering, Guangxi University of Science and Technology, Liuzhou 545006, China

**Keywords:** three-dimensional overall jagged internal finned tube, heat transfer enhancement, experiment, numerical simulation

## Abstract

To satisfy the demand for efficient heat transfer, a novel three-dimensional overall jagged internal finned tube (3D-OJIFT) was fabricated, using the rolling–ploughing/extruding method. The thermal performance of the 3D-OJIFT were studied and compared in experiments and three-dimensional numerical simulations. The RNG *k*-*ε* turbulence model is well verified with the experimental results. By analyzing the distributions of velocity, temperature, and turbulence kinetic energy, it was found that the 3D-OJIFT destroyed the development of the velocity and thermal boundary layers, increased the turbulence disturbance, and reduced the temperature gradient, thus improving the heat transfer. The influences of the jagged height and jagged spiral angle of the 3D-OJIFT are discussed. The Nu and f increased as the jagged height of the 3D-OJIFT increased. The Nusselt number of the 3D-OJIFT was 1.67–2.04 times the value for the smooth tube. In addition, the comprehensive heat transfer performance of the 3D-OJIFT improved after increasing the jagged spiral angle. Compared with conventional internal helical-finned tubes and other reinforcement structures reported in the literature, the 3D-OJIFT demonstrated better comprehensive heat transfer performance. Finally, empirical correlations of the 3D-OJIFT were obtained.

## 1. Introduction

Shell-and-tube heat exchangers have been widely applied in many fields, such as air conditioning, power plants, solar heaters, and microreactors [1]. To reduce energy consumption and improve heat transfer, different enhanced heat transfer technologies including passive and active methods have been studied and used in the past few decades [2]. Passive methods typically utilize insertions and/or modified surfaces, which increase heat transfer as the friction factor increases. Finned tubes effectively improve heat transfer with small friction factors and are easy to manufacture and maintain. Owing to these characteristics, finned tubes have been widely used in shell-and-tube heat exchangers.

Many experiments have been conducted to study the heat transfer performance of the tube side of finned tubes. Among these enhanced heat transfer technologies, dimpled tubes, corrugated tubes, internal helical-finned tubes (IHFTs), and three-dimensional internal finned tubes (3D-IFTs) have been investigated experimentally [3]. Dimpled tubes have high heat-transfer efficiency and a relatively small friction factor, owing to the swirls and vortices generated by the dimples [4,5]. The effects of different parameters, such as the concavity, dimple imprint diameter, and depth on the thermal performance of dimpled tubes were investigated [6]. The results of those studies indicated that the heat transfer enhancement could reach 40% with negligible pressure drop [7]. Xie et al. [8] investigated the effects of the number of starts, radius, spiral pitch, depth, and transverse length of a helically dimpled tube on its thermal-hydraulic performance. The correlations for Nu and f were obtained. Corrugated tubes have also been used for improving the thermal performance of the tube side, owing to the swirls induced by corrugations and a larger heat transfer area. Different helical pitches, heights, and characteristics of helical corrugated tubes have been investigated [9,10]. All of the studied corrugated geometries performed better than smooth tubes, but corrugated tubes exhibited worse thermal performance in laminar flows [10,11]. IHFTs are effective at improving the thermal performance of the tube side [12]. Ma et al. [13]. reported that the j-factor for an IFT was approximately 3.5 times that of a smooth tube. The effects of different structure parameters (fin number, diameter, helix angle, width, and fin height) on the heat transfer performance were studied. Lie et al. [14]. found that Nu and f both increased as the values of these parameters increased and that the helical angle had the strongest effect on the heat transfer enhancement. Celen et al. [15]. developed a friction equation for assessing the friction performance. IHFTs have been widely used. However, owing to the low fin height of IHFTs, the spiral flow in such tubes limits the adjustment of the boundary layer [12]. Therefore, IHFTs cannot satisfy the demand for efficient heat transfer.

To address the need for efficient heat transfer, 3D-IFTs have attracted considerable attention in industry, owing to their higher fins. Wang et al. [16] prepared a twisted pin-finned pipe and circumferential pin-finned pipe based on the 3D printing method, and the results showed that the heat transfer coefficients were larger than those of a corresponding plain pipe. However, the productivity and manufacturing costs of these tubes do not meet the requirements of producers. Huang et al. [17] proposed arc-shaped inner-finned tubes and experimentally analyzed their heat transfer performance. However, the heat transfer characteristics and other structural parameters of the heat transfer performance were not addressed. Han et al. [18] studied the heat transfer performance of 3D-IFTs with porous copper fiber inserts. The effects of the diameter, spacing, and porosity of the porous copper fiber inserts on the thermal performance were investigated.

According to the aforementioned literature review, relatively little research has been conducted on 3D-IFTs. The flow inside 3D-IFTs remains poorly understood. In addition, a few empirical correlations have been considered for predicting the thermal performance of 3D-IFTs. In this study, a novel three-dimensional overall jagged internal finned tube (3D-OJIFT) was developed based on the rolling–ploughing/extruding (RPE) method. The characteristics of the 3D-OJIFT were analyzed. Thermal performance experiments were conducted, and the Nu and f experimental values were used for validating the numerical model. By analyzing the distributions of the turbulent kinetic energy, velocity field, temperature field, and local convective heat transfer coefficient, the mechanisms of the 3D-OJIFT heat transfer enhancement were elucidated. Moreover, the effects of the jagged height and jagged spiral angle on the 3D-OJIFT thermal performance were investigated. The 3D-OJIFT was compared with conventional IHFTs. Finally, the empirical correlations for Nu and f were computed and presented.

## 2. Experimental Studies

### 2.1. Characterization of the 3D-OJIFT

**Figure 1:**
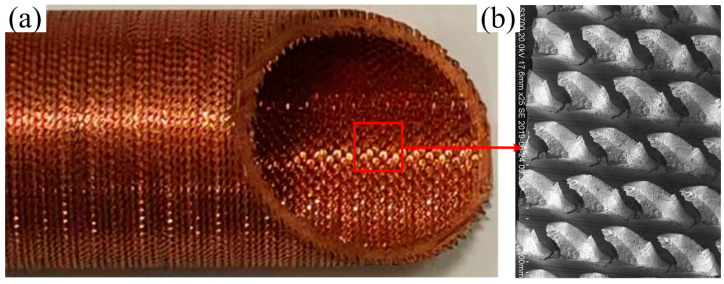
The novel 3D-OJIFT manufactured using the RPE method: (**a**) photographs of 3D-OJIFT, (**b**) SEM of the 3D-OJIF.

To satisfy the demand for efficient heat transfer, a 3D-OJIFT was fabricated using the RPE method. The manufacture of a 3D-OJIFT involves two processes. First, the inner thread structure is formed inside the tube by a rolling process, and then, the inner thread structure is ploughed and extruded by using the self-designed tool. Finally, 3D-OJIFTs are obtained. As shown in Figure 1, the novel internal fin has a jagged structure with the characteristics of a three-dimensional integral and rough surface. The 3D-OJIFT comprises a jagged internal fin and staggered grooves, distributed at a certain spiral angle. Compared with conventional IHFTs, the internal fin height of the 3D-OJIFT is significantly higher, which is conducive to increasing the heat transfer area and turbulent flow.

### 2.2. Experimental System

**Figure 2:**
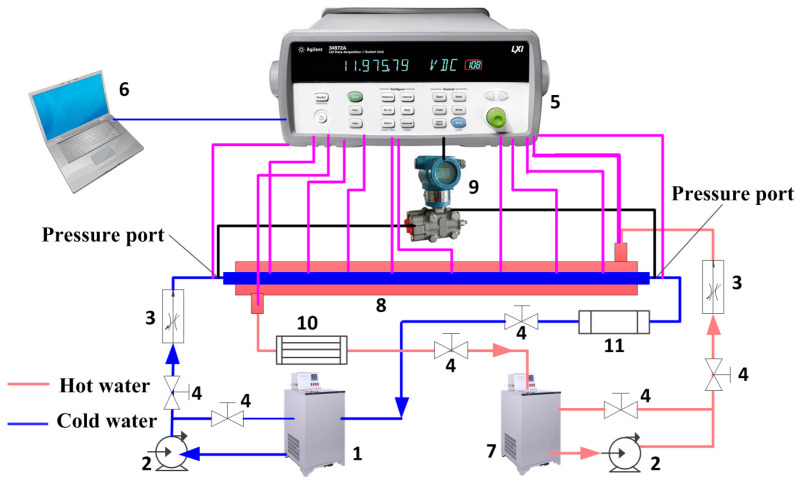
Schematic of the experimental system: (1) cold water tank; (2) pump; (3) rotameter; (4) control valve; (5) data acquisition system; (6) laptop; (7) hot water tank; (8) two coaxial tubes; (9) pressure test element; (10) thermal compensation element; (11) fan coil unit.

As shown in Figure 2, the experimental device, which was described in detail in our previous study [19], consisted of two coaxial tubes, a hot water circulation loop, a cold water circulation loop, a data-acquisition system, a temperature measuring element, and a pressure measuring element. The two coaxial tubes consist of a plexiglass outer pipe and a copper pipe. The geometric parameters of the plexiglass outer tube are 1800 mm in length, 45 mm in outer diameter, and 5 mm in wall thickness. The geometric parameters of the smooth tube in the test section are as follows: the length is 2000 mm, the inner diameter is 19 mm, and the wall thickness is 1.5 mm. The geometric parameters of the 3D-OJIFT are 2000 mm in length, 22 mm in outer diameter, and 0.8 mm in jagged height. Cold fluid flowed inside the tube, whereas hot fluid on the shell side flowed in the opposite direction. The flow rates of fluid were measured using a rotary flowmeter and regulated using a control valve. The cold water inlet temperature is 25 °C, the hot water inlet temperature is 77 °C, and the inlet temperature was adjusted using compensators and temperature controllers on the water tank. Eight T-type thermocouples were evenly distributed on the outer surface of the internal tube wall for measuring the temperature of the outer tube wall. Four T-type thermocouples were located at the inlet and outlet of the hot and cold water circulation loops to measure water temperature, and the accuracy of these T-type thermocouples was ±0.1 °C. The pressure difference between the pipe inlet and outlet was measured using a pressure transmitter (accuracy, ±0.5%). The experimental data of the temperature and pressure were collected using a data acquisition system. The pipeline and two coaxial tubes were insulated to prevent heat loss.

### 2.3. Data Simplification and Uncertainty Analysis

The main objective was to obtain the *Nu* and *f* values for the tube side. The method and steps were as follows:

The heat transfer rates (HTRs) of hot and cold water were calculated as

(1) $$Q_{c}=\overset{•}{m_{c}}c_{p}(t_{c,o}-t_{c,i})$$

(2) $$Q_{h}={\overset{•}{m}}_{h}c_{p}(t_{h,i}-t_{h,o})$$

Considering the heat balance of the HTR, the average HTR was considered to be the HTR of the test section.

(3) $$Q_{\mathrm{ave}}=\frac{Q_{c}+Q_{h}}{2}$$

The overall heat transfer coefficient was calculated as

(4) $$U=\frac{Q_{ave}}{A_{i}\Delta T_{m}}$$

where $\Delta T_{m}$ denotes the logarithmic mean temperature difference. The overall heat transfer coefficient was expressed as follows:

(5) $$\frac{1}{U}=\frac{1}{h_{i}}+R_{w}+\frac{A_{i}}{h_{o}A_{o}}+R_{f}$$

where *R_f_* is the fouling resistance and *R_w_* is the conduction resistance.

The heat transfer correlation *h_o_* for the annulus side was calculated as follows [20]:

(6) $$Nu_{o}=0.023Re^{0.8}Pr^{0.4}$$

(7) $$h_{o}=\frac{k}{D_{H}}0.023Re^{0.8}Pr^{0.4}$$

where *k* is the thermal conductivity of the annulus and *D_H_* represents the hydraulic diameter of the annulus.

The *Nu* for the tube side was given by

(8) $$Nu_{i}=\frac{h_{i}d_{i}}{k}$$

where *d_i_* is the inside nominal diameter of the tube [21].

The *Re* and *f* were calculated as follows:

(9) $$Re=\frac{\rho \nu d_{H}}{\mu }$$

(10) $$f=\frac{2\Delta pd_{i}}{l\rho \nu^{2}}$$

The performance evaluation criterion (*PEC*) was used [22]:

(11) $$PEC=\frac{Nu/Nu_{0}}{{\left(f/f_{0}\right)}^{1/3}}$$

where *Nu*_0_ and *f*_0_ are the Nusselt number and friction factor of the smooth tube, respectively.

Based on the method of Schultz and Cole [23], the maximal uncertainties of the *Re*, *f*, nd *Nu* were 1.19%, 2.32%, and 1.66%, respectively.

### 2.4. Experimental Results

To ensure the accuracy of the experimental data, *Nu* and *f* were measured in smooth pipes and compared with the empirical correlations. The Gnielinski correlation [24] was defined as

(12) $$Nu=\frac{(Re-1000)(f/8)Pr}{1+12.7(Pr^{2/3}-1){\left(f/8\right)}^{0.5}}$$

The Petukhov correlation [25] was defined as

(13) $$f={\left(0.79\ln Re-1.64\right)}^{-2}$$

**Figure 3:**
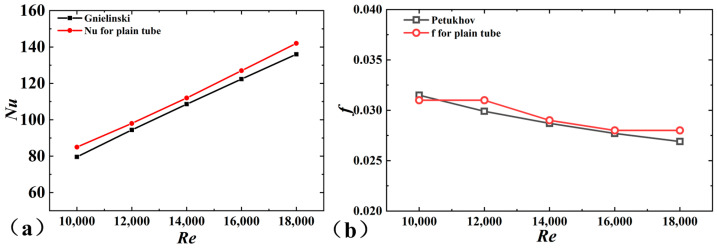
Comparison of the experimental results for a smooth tube and empirical correlations: (**a**) *Nu*, (**b**) *f*.

As shown in Figure 3, the experimental results agreed well with those empirical results. The maximal error of *Nu* was approximately 6.7%, while the error of *f* was approximately 4%. Based on the experimental platform, the thermal performance of the 3D-OJIFT was tested, and *Nu* and *f* of the 3D-OJIFT are depicted in Figure 3.

## 3. Numerical Simulations

### 3.1. Physical Model

**Figure 4:**
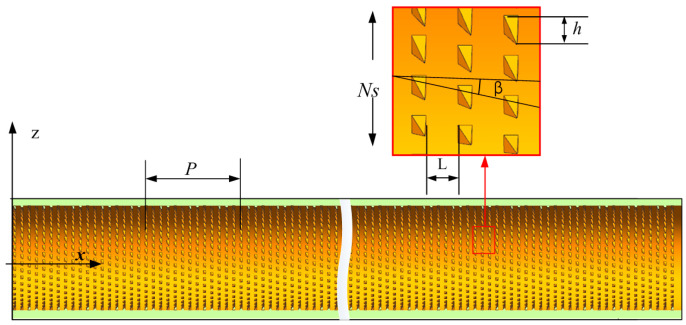
Schematic of the 3D-OJIFT.

The physical model of the 3D-OJIFT is shown in Figure 4. The wall thickness was 1.5 mm, and the period length (P) of the 3D-OJIFT ranged from 20 mm to 140 mm, which was defined as the length of the calculation domain. The three-dimensional overall jagged internal fin (3D-OJIF) was characterized by the jagged height, jagged spiral angle, and number of 3D-OJIFs. The effects of varying the jagged height (*h* = 0.4 mm, 0.6 mm, and 0.8 mm) and jagged spiral angle (*β* = 22°, 42°, and 65°) on the thermal performance were studied.

### 3.2. Governing Equations

A three-dimensional computational fluid dynamics (CFD) model of the thermal performance in the 3D-OJIFT was presented. The assumptions, which were commonly made in many studies [26,27], were as follows: (1) the flow is isotropic, continuous, stable, and incompressible and has constant physical properties; (2) the effect of gravity is negligible. The equations were as follows:

Continuity equation:

(14) $$\frac{\partial (\rho u_{i})}{\partial x_{i}}=0$$

Momentum equation:

(15) $$\frac{\partial (\rho u_{i}u_{j})}{\partial x_{j}}=-\frac{\partial p}{\partial x_{i}}+\frac{\partial }{\partial x_{j}}\left[u\left(\frac{\partial u_{i}}{\partial x_{j}}+\frac{\partial u_{j}}{\partial x_{i}}\right)\right]-\frac{\partial \left(\rho \bar{u_{i}^{\prime }u_{j}^{\prime }}\right)}{\partial x_{j}}$$

Energy equation:

(16) $$\frac{\partial (u_{j}(\rho e+p))}{\partial x_{j}}=\frac{\partial }{\partial x_{j}}(\lambda \frac{\partial T}{\partial x_{j}})$$

where *ρ* and *c_p_* are the fluid density and fluid specific heat, respectively, *p* and *λ* are the pressure and thermal conductivity, respectively, and *T* and *u* denote the temperature and fluid viscosity, respectively.

The RNG *k*-*ε* turbulence model [28,29] was employed in this paper.

(17) $$\frac{\partial (\rho ku_{i}))}{\partial x_{j}}=\frac{\partial }{\partial x_{j}}\left(\left(\mu +\frac{\mu_{t}}{\sigma_{k}}\right)\lambda \frac{\partial k}{\partial x_{j}}\right)+C_{1\varepsilon }\frac{\varepsilon }{k}G_{k}-C_{2\varepsilon }\frac{\varepsilon^{2}}{k}\rho$$

(18) $$\frac{\partial (\rho \varepsilon u_{i})}{\partial x_{j}}=\frac{\partial }{\partial x_{j}}\left(\lambda \frac{\partial k}{\partial x_{j}}\left(\mu +\frac{\mu_{t}}{\sigma_{k}}\right)\right)+C_{1\varepsilon }\frac{\varepsilon }{k}G_{k}-C_{2\varepsilon }\rho \frac{\varepsilon^{2}}{k}$$

### 3.3. Mesh and Boundary Conditions

**Figure 5:**
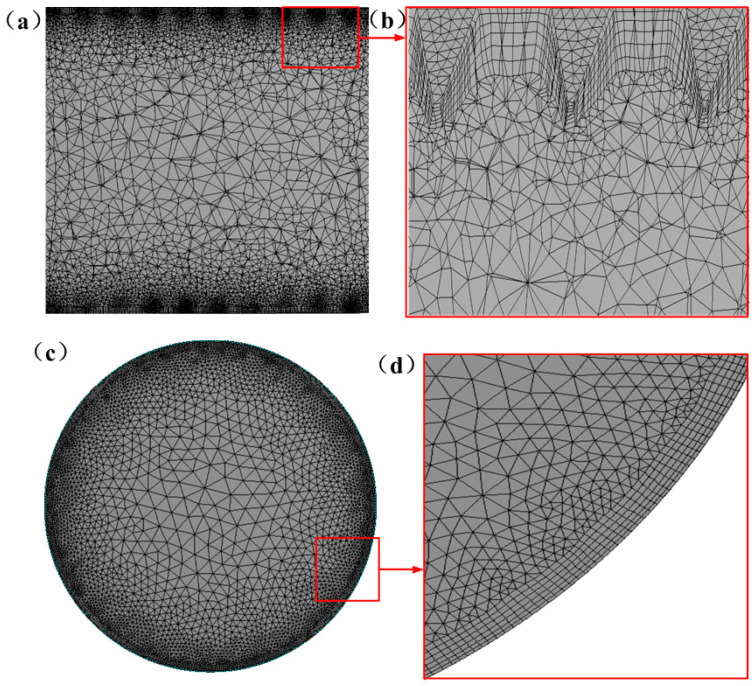
The fluid domain mesh: (**a**,**b**) in the y = 0 plane; (**c**,**d**) in the x = 0 plane.

As shown in Figure 5, an unstructured mesh was used for discretizing the computational domain. Local mesh refinement was applied to the surface of the 3D-OJIFT, as shown in Figure 5b. The grid independence at a Reynolds number of 14,000 was investigated for the 3D-OJIFT using four sets of meshes (2,493,659, 3,382,197, 4,283,296, and 5,115,893). The relative difference between the Nusselt numbers for the 4,283,296 and 5,115,893 grids was within 3%, and the relative difference between the friction coefficients was within 3%. Thus, the mesh independence analysis indicated that the 4,283,296 grid was sufficient for capturing the characteristics of the flow; thus, this grid was used in subsequent calculations.

Considering the repetitive periodic geometry of the 3D-OJIFT and the computational cost, periodic boundary conditions were used, which have been adopted by much of the literature [30]. Periodic conditions were imposed on the inlet and outlet. The constant temperature condition (350 K) was used for the 3D-OJIFT wall. The upstream temperature was 298 K. No-slip conditions were prescribed for the surfaces of the 3D-OJIFT walls. The working fluid is water. The Reynolds number was in the 10,000–18,000 range. The finite-volume method was implemented for solving the governing equations. The second-order upwind algorithm was used for discretizing the momentum and energy terms. The standard scheme was employed for the pressure term. The convergence criterion was 10^−8^ for energy, while 10^−6^ was used for other equations.

### 3.4. Data Reduction

After convergence, the thermo-hydraulic performance was profiled. The convective heat transfer coefficient (*h_t_*) of the tube wall was

(19) $$h_{t}=\frac{q}{T_{m,w}-T_{m,f}}$$

where *q* and $T_{m,w}$ denote the heat flux of the pipe wall and the mean temperatures pipe wall, respectively, and $T_{m,f}$ denotes the mean temperatures of the fluid. $T_{m,w}$ and $T_{m,f}$ were defined as follows:

(20) $$T_{m,w}=\frac{1}{A}\int_{0}^{A}T_{w}dS$$

(21) $$T_{m,f}=\frac{\int_{0}^{D/2}ru_{x}rT_{f}dr}{\int_{0}^{D/2}ru_{x}rdr}$$

where $u_{x}$ and $T_{w}$ are the axial velocity of the cross-section and the temperature of the pipe wall, respectively. $T_{f}$ denotes the temperature of the water, and *A* is the surface area of the pipe wall.

### 3.5. Validation

**Figure 6:**
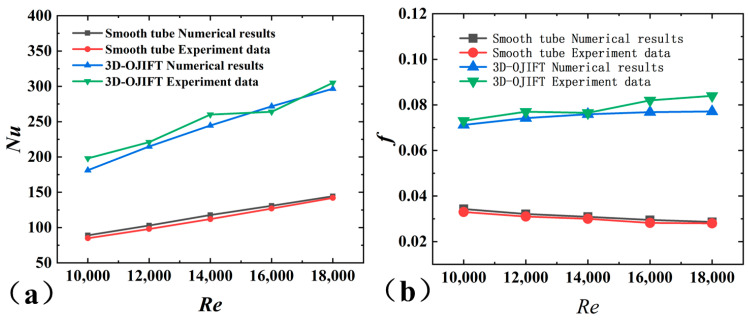
Comparison of numerical results and experimental data: (**a**) *Nu*; (**b**) *f*.

The RNG *k-ε* turbulence model was first verified. As depicted in Figure 6, the numerical results for smooth tubes are compared with the experimental results. The maximal errors of Nusselt number and friction factor are below 4.9% and 5%, respectively. In addition, the numerical results for the 3D-OJIFT with *β* = 22° and *h* = 0.8 mm were compared with the corresponding experimental results. The data showed that the maximal deviation of *Nu* was 9.2%, while the maximal deviation of *f* was 8.9%. Thus, the numerical calculation data agreed well with the experimental results.

## 4. Results and Discussion

### 4.1. Thermo-Hydraulic Characteristics

Considering the distributions of velocity, temperature, turbulent kinetic energy (*TKE*), and the local convective heat transfer coefficient as examples, the thermo-hydraulic characteristics of the 3D-OJIFT were analyzed. The *TKE* quantitatively describes the turbulence fluctuations of the water in a tube and was defined as

(22) $$TKE=\frac{\bar{\mu_{z}^{\prime 2}}+\bar{\mu_{y}^{\prime 2}}+\bar{\mu_{x}^{\prime 2}}}{2}$$

where $\mu_{i}^{\prime }$ denotes the velocity fluctuation. Compared with smooth tubes, the better thermal performance was mainly owing to the following.

**Figure 7:**
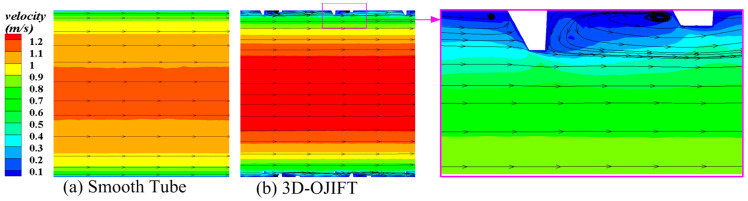
Axial velocity contours of the 3D-OJIFT and smooth pipe at y = 0.2 mm: (**a**) smooth tube; (**b**) 3D-OJIFT.

Figure 7 shows the axial flow field for the 3D-OJIFT and the corresponding smooth tube at the y = 0.2 mm section. The axial velocity field of the 3D-OJIFT differs from that of the smooth tube. The axial velocity is low near the 3D-OJIFT tube wall, whereas it is high at the center of the 3D-OJIFT tube wall. Evidently, the 3D-OJIF configuration strongly affects axial velocity, yielding a more mixed flow, secondary flow, and swirl flow in the 3D-OJIFT, thus improving the heat transfer performance.

**Figure 8:**
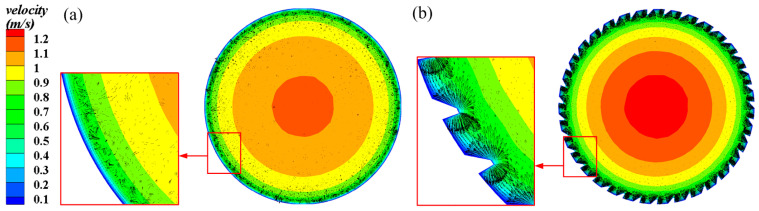
Radial velocity contours of the smooth tube and 3D-OJIFT at x = 8.5 mm: (**a**) smooth tube; (**b**) 3D-OJIFT.

Figure 8 depicts the radial velocity fields of the 3D-OJIFT and smooth tube at the x = 8.5 mm section. As expected, the 3D-OJIF configuration strongly affects the flow field. As the fluid flows through the 3D-OJIFT, the flow path inside the 3D-OJIFT shrinks owing to the 3D-OJIF surface, causing the velocity to increase. With periodic variation in the velocity field, momentum exchange improves fluid mixing and vortex generation between the mainstream region and the 3D-OJIF region.

**Figure 9:**
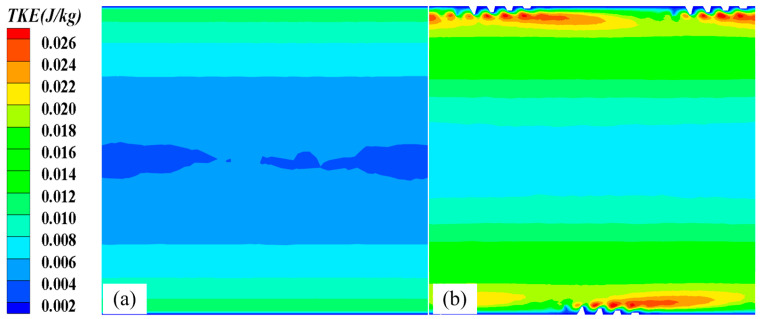
TKE distributions of the smooth tube and 3D-OJIFT at x = 8.5 mm: (**a**) smooth tube; (**b**) 3D-OJIFT.

Figure 9 compares the TKE distributions of the 3D-OJIFT and the smooth tube. Evidently, the TKE of the 3D-OJIFT is significantly stronger than that of the smooth tube, owing to the axial flow disturbance near the jagged fin surface. This improves the circulation flow and fluid mixing between the 3D jagged fin surface and the core region, resulting in the destruction of the thermal boundary layer near the jagged fin surfaces. In addition, vortices occur near the jagged fin surface. This may intensify high turbulence, significantly increasing the *Nu* and *f* values of the fluid flowing in the 3D-OJIFT.

**Figure 10:**
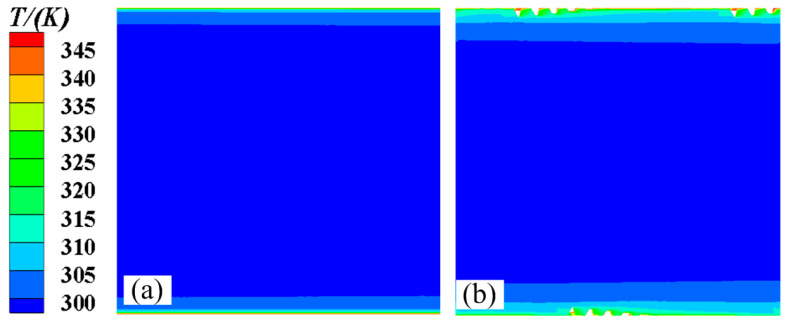
Axial temperature contours of the smooth tube and 3D-OJIFT at x = 8.5 mm: (**a**) smooth tube; (**b**) 3D-OJIFT.

**Figure 11:**
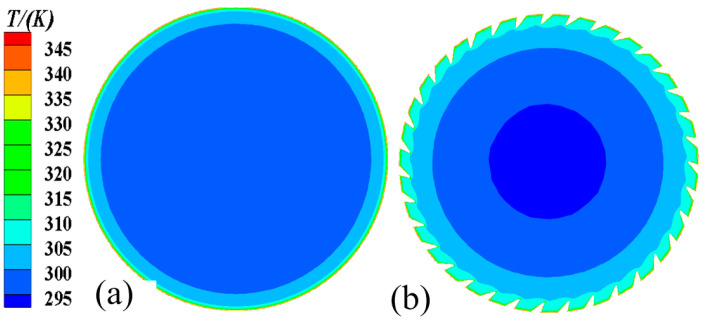
Radial temperature contours of the smooth tube and 3D-OJIFT at y = 0.2 mm: (**a**) smooth tube; (**b**) 3D-OJIFT.

**Figure 12:**
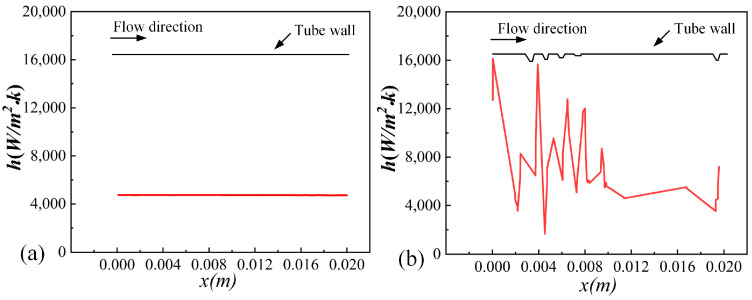
Distributions of the convective heat transfer coefficient: (**a**) smooth tube, (**b**) 3D-OJIFT.

Figure 10 and Figure 11 show the axial and radial temperature distributions for the smooth tube and 3D-OJIFT, respectively. As shown in Figure 10 and Figure 11, the temperature gradient in the inner wall region of the 3D-OJIFT is steeper than that in the smooth tube. In addition, the 3D-OJIFT precludes the thermal boundary layer from full development. Thus, using a jagged internal fin can improve heat transfer. Figure 12 shows the local heat transfer coefficient for the tube wall along the X direction. Compared with the smooth tube, the local heat transfer coefficient of the 3D-OJIFT is larger and more variable. Therefore, the 3D-OJIFT exhibits better heat transfer performance.

### 4.2. The Effect of the Jagged Height

**Figure 13:**
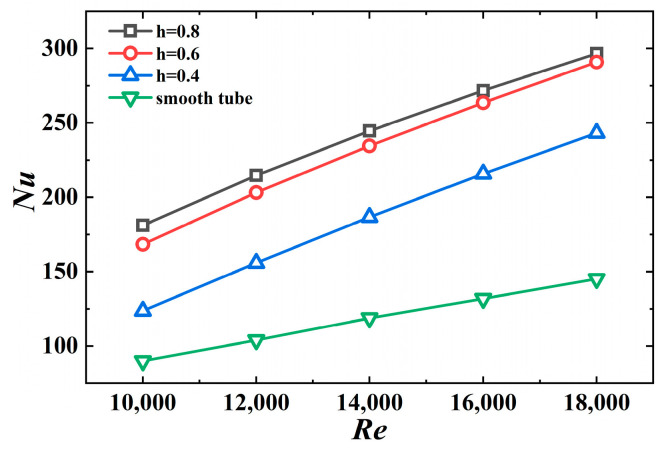
*Nu* versus *Re* for 3D-OJIFTs with different *h*.

Figure 13 shows the Nusselt number versus the Reynolds number for different jagged heights. Evidently, the Nusselt number of the 3D-OJIFT increases with increasing jagged height. The results for the 3D-OJIFT with *h* = 0.8 mm are better than for the other cases, with approximately a 2.06-fold improvement over the value for the smooth tube at *Re* = 12,000. The *Nu* value at *h* = 0.6 mm is 1.87–2.0 times that for the smooth tube. The results suggest that the 3D-OJIFT increases the thermal boundary layer disruption of turbulence; thus, the 3D-OJIFT exhibits better thermal performance compared with the smooth tube.

**Figure 14:**
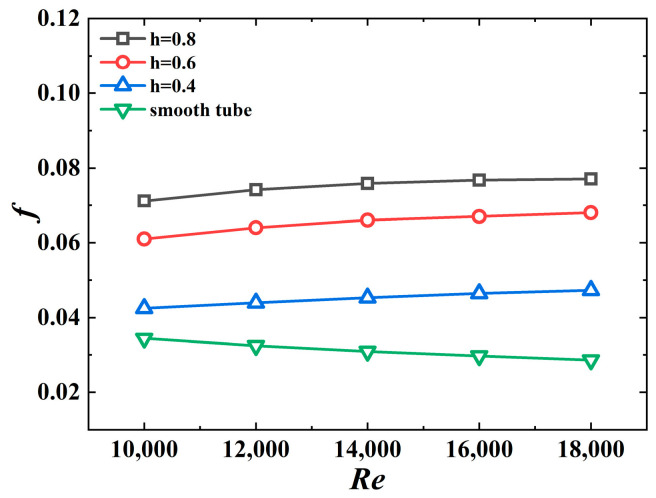
*f* versus *Re* for 3D-OJIFTs with different *h*.

The effect of the jagged height on the friction coefficient of the 3D-OJIFT is shown in Figure 14. The results suggest that the friction coefficient increases as the jagged height increases. For *h* = 0.8 mm, the *f* value is the largest, 2.06–2.69 times the value for the smooth tube. This is owing to the increased height of the fin, increased contact area between the fin and fluid, and greater flow obstruction of the tube, resulting in a greater *f.*

**Figure 15:**
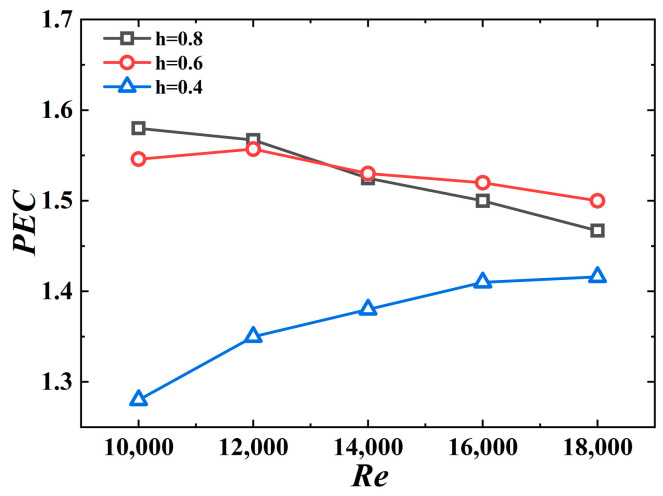
*PEC* versus *Re* for 3D-OJIFTs with different *h*.

Figure 15 shows the PEC values for different jagged height values. The PEC values are in the 1.28–1.58 range, indicating that the 3D-OJIFT exhibits better comprehensive heat transfer performance. Another conclusion to be drawn from Figure 15 is that the 3D-OJIFTs with *h* = 0.6 mm and *h* = 0.8 mm perform better than the 3D-OJIFT with *h* = 0.4 mm. For *Re* above 14,000, the comprehensive heat transfer performance of the 3D-OJIFT with *h* = 0.6 mm is better than that of the 3D-OJIFT with *h* = 0.8 mm. Therefore, it is important to choose an appropriate height for the inner fin of the 3D-OJIFT.

### 4.3. The Effect of the Jagged Spiral Angle

**Figure 16:**
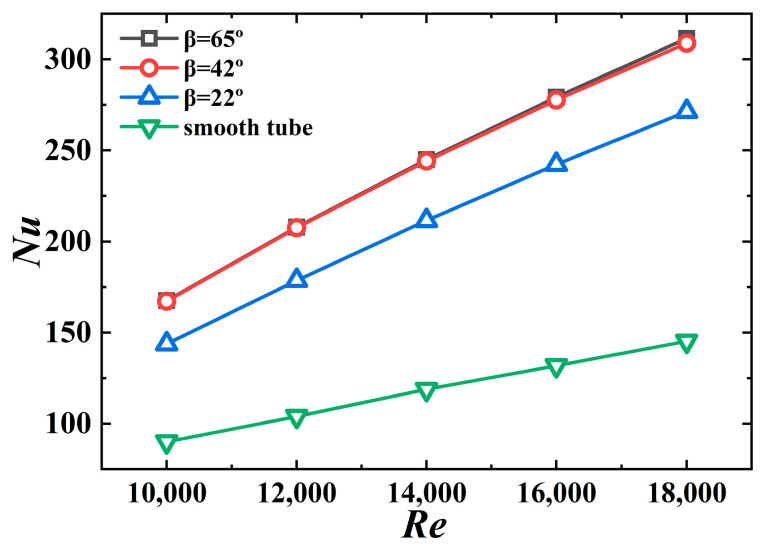
*Nu* versus *Re* for 3D-OJIFTs with different β.

Figure 16 shows the Nusselt number for different jagged spiral angle values. The Nusselt number of the 3D-OJIFT increases as the jagged spiral angle increases. The results for *β* = 22° and *β* = 65° are better than 1.60–1.87-fold and 1.86–2.14-fold, respectively, compared with the corresponding results for the smooth tube. Because a wider jagged spiral angle is conducive to generating a secondary flow of fluid and increasing the turbulence of the fluid, wider jagged spiral angles are associated with better heat transfer performance. However, for the jagged spiral angles exceeding 42°, *Nu* does not increase significantly. Figure 16 shows that the *Nu* values for the jagged spiral angles of 42° and 65° are almost the same. This is because when the helix angle exceeds 42°, the disturbance of the two spiral-angle structures to the boundary layer is limited.

**Figure 17:**
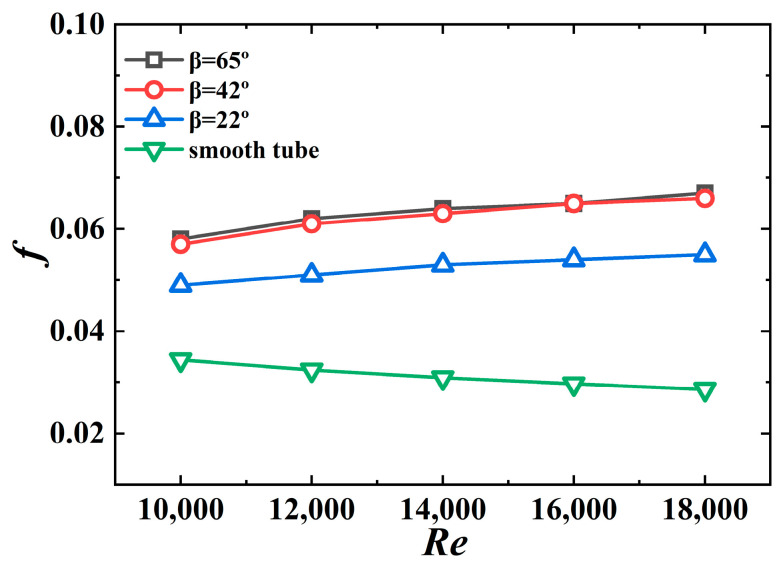
*f* versus *Re* for 3D-OJIFTs with different *β*.

Figure 17 shows the effect of the jagged spiral angle on the friction coefficient of the 3D-OJIFT. Evidently, the wider the jagged spiral angle of the 3D-OJIFT, the larger the friction coefficient. For *β* = 22°, the friction coefficient of the 3D-OJIFT is 1.42–1.92 times that for the smooth tube. For *β* = 65°, the friction coefficient is 1.67–2.34 times that for the smooth tube. A wide jagged spiral angle increases the area of the 3D-OJIFT exposed to the incoming flow, and the change in the fluid flow direction also increases, implying that the obstruction to the incoming flow increases. Therefore, 3D-OJIFTs with wider jagged spiral angles exhibit more friction.

**Figure 18:**
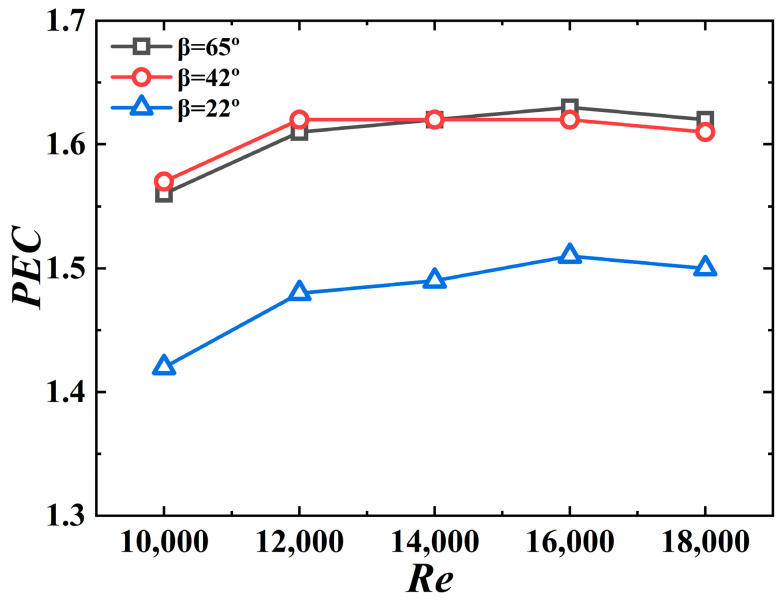
*PEC* versus *Re* for 3D-OJIFTs with different *β*.

Figure 18 shows *PEC* versus the *Re* number for different jagged spiral angles. The *PEC* value for the 3D-OJIFT with the above jagged spiral angle exceeds 1 (range, 1.42–1.63), implying that the 3D-OJIFT exhibits good comprehensive heat transfer performance. In addition, 3D-OJIFTs with wider jagged spiral angles should be used for ensuring better comprehensive heat transfer performance at high *Re*.

### 4.4. Comparison with the Conventional Two-Dimensional Spiral Fin Tubes

**Figure 19:**
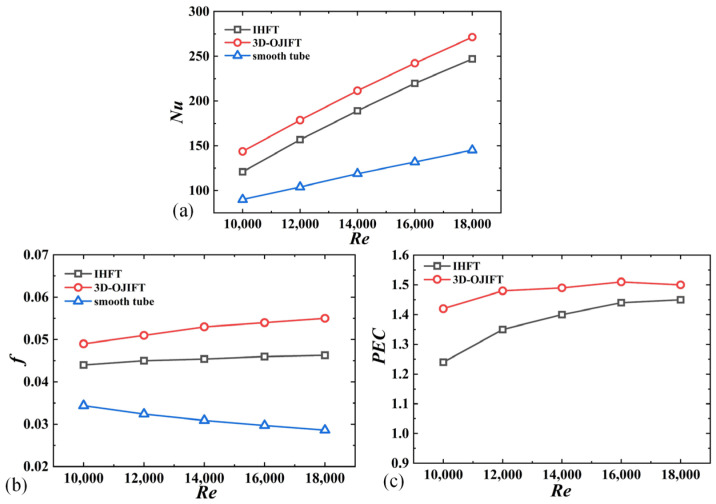
Comparison of the thermal performances of the conventional IHFT and designed 3D-OJIFT for *Re* ranging from 10,000 to 18,000: (**a**) *Nu*; (**b**) *f*; (**c**) *PEC*.

A conventional IHFT was selected for comparison to evaluate the thermal performance of the designed 3D-OJIFT. The geometric parameters of the IHFT were as follows: the fin height was *h* = 0.3 mm, and the helix angle was *β* = 22°. The helix angles of the 3D-OJIFT and IHFT were the same. The height of the 3D-OJIF was 0.6 mm, and fin spacing (L) was 0.6 mm. As shown in Figure 19a, the 3D-OJIFT had a larger *Nu*. The *Nu* of the 3D-OJIFT was approximately 1.87 times that of the smooth tube and 1.1 times that of the IHFT at *Re* = 18,000. As shown in Figure 19b,c, although the friction coefficient of the 3D-OJIFT was larger than that of the conventional IHFT (at 1.39–1.47 times that of the conventional IHFT), the designed 3D-OJIFT exhibited better comprehensive heat transfer performance. The *PEC* value of the 3D-OJIFT was 1.03–1.15 times that of the conventional IHFT.

### 4.5. Comparison with Other Reported Studies

To better evaluate the heat transfer performance of the novel 3D-OJIFT, it was compared with other enhancement techniques reported in the literature, as shown in Table 1. The maximum *Nu*/*Nup* of the 3D-OJIFT is slightly higher than that of other enhancement technologies. However, the *f*/*fp* of the 3D-OJIFT is much smaller than those of an internal helically-finned tube [31], corrugated tube [32], and dimpled tube [33]. The maximum PEC of the 3D-OJIFT is increased by 14.4%, 32.7%, and 16.1%, respectively. Therefore, compared with the other four reinforcement structures reported in the literature, the new 3D-OJIFT presents the best overall heat transfer performance.

### 4.6. Correlations of the Nusselt Number and Friction Factor

**Figure 20:**
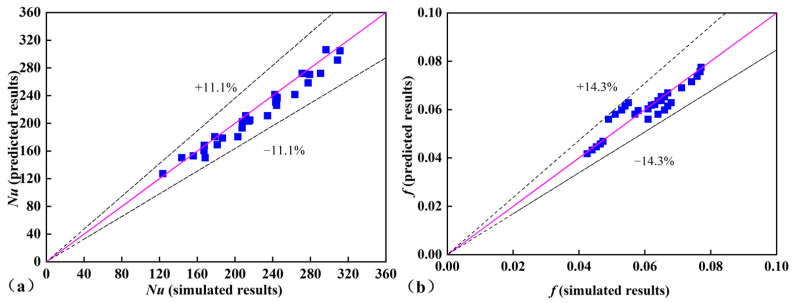
Discrepancies between the numerical and simulated values of *Nu* and *f*: (**a**) *Nu*, (**b**) *f*.

The empirical correlations for the 3D-OJIFT were obtained, as defined by Equations (23) and (24). These empirical Equations (23) and (24) are valid for the following ranges: (10,000 ≤ *Re* ≤ 18,000, 0.4 mm ≤ *h* ≤ 0.8 mm, 22° ≤ *β* ≤ 65°). Figure 20 shows that the Nu deviation between the predicted results and simulated data was approximately ±11.1%, and the corresponding deviation for *f* was approximately ±14.3%. Therefore, the empirical equations are suitable for predicting *Nu* and *f*.

(23) $$Nu=0.012039Re^{1.011559}h^{0.40981}\beta^{0.10465}$$

(24) $$f=0.011077Re^{0.19686}h^{0.7253}\beta^{0.05752}$$

## 5. Conclusions

The thermo-hydraulic characteristics of a novel 3D-OJIFT were studied and compared using experimental and numerical methods. The main conclusions are as follows.

1. A novel 3D-OJIFT was fabricated using the RPE method. The 3D-OJIF increased the heat transfer area, prevented the development of the velocity and thermal boundary layers, increased the turbulence disturbance, and reduced the temperature gradient, thus enhancing the heat transfer.
2. The jagged height of the 3D-OJIFT importantly affected the thermal performance. The *Nu* and *f* both increased as the jagged height increased. The *Nu* of the 3D-OJIFT was 1.59–2.14 times that of the smooth tube.
3. The jagged spiral angle of the 3D-OJIFT was very sensitive to the thermal performance. Both *Nu* and *f* increased as the jagged spiral angle increased. For better comprehensive heat transfer performance, 3D-OJIFTs with wider jagged spiral angles should be chosen at high Re.
4. Compared with conventional IHFTs and other reported studies, the proposed 3D-OJIFT exhibited better comprehensive heat transfer performance. The *PEC* value of the proposed 3D-OJIFT was 1.03–1.15 times that of the conventional IHFT. Empirical correlations were developed to predict the *Nu* and *f* values for 3D-OJIFTs. The discrepancies for *Nu* and *f* were within ±11.1% and ±14.3%, respectively.

## Figures and Tables

**Table 1:** Comparison between the present study and other reported studies.

Authors	Enhanced Structures	*Nu/Nup*	*f/fp*	*PEC*	*Re*
Present study	3D-OJIFT	2.01–2.04	2.01–2.69	1.46–1.58	10,000–18,000
Li et al. [31]	Internal helically finned tube	1–2.0	1–3.0	1–1.38	2500–15,000
Dizaji et al. [32]	Corrugated tube	1.5–1.75	3.52–3.48	0.98–1.19	7000–17,500
Vicente et al. [33]	Dimpled tube	1.5–2.1	3.2–4.2	1.01–1.36	2000–19,000

## Data Availability

All data to support the results of this study are included in this article.

## References

[1] Khatibi M., Kowsari M.M., Golparvar B., Niazmand H. (2021). Optimum loading of aluminum additive particles in unconsolidated beds of finned flat-tube heat exchangers in an adsorption cooling system. Appl. Therm. Eng..

[2] Tiwari A.K., Javed S., Oztop H.F., Said Z., Pandya N.S. (2021). Experimental and numerical investigation on the thermal performance of triple tube heat exchanger equipped with different inserts with WO3/water nanofluid under turbulent condition. Int. J. Therm. Sci..

[3] Ji W.-T., Jacobi A.M., He Y.-L., Tao W.-Q. (2015). Summary and evaluation on single-phase heat transfer enhancement techniques of liquid laminar and turbulent pipe flow. Int. J. Heat Mass Transf..

[4] Kukulka D.J., Smith R., Li W. (2015). Comparison of tubeside condensation and evaporation characteristics of smooth and enhanced heat transfer 1EHT tubes. Appl. Therm. Eng..

[5] Li M., Khan T.S., Al-Hajri E., Ayub Z.H. (2016). Single phase heat transfer and pressure drop analysis of a dimpled enhanced tube. Appl. Therm. Eng..

[6] Kim J., Doo J., Ha M., Yoon H., Son C. (2012). Numerical study on characteristics of flow and heat transfer in a cooling passage with protrusion-in-dimple surface. Int. J. Heat Mass Transf..

[7] Afanasyev V.N., Chudnovsky Y.P., Leontiev A.I., Roganov P.S. (1993). Turbulent flow friction and heat transfer characteristics for spherical cavities on a flat plate. Exp. Therm. Fluid Sci..

[8] Xie S., Guo Z., Gong Y., Dong C., Liu J., Ren L. (2022). Numerical investigation of thermal-hydraulic performance of a heat exchanger tube with helical dimples. Int. J. Therm. Sci..

[9] Hu Q., Qu X., Peng W., Wang J. (2022). Experimental and numerical investigation of turbulent heat transfer enhancement of an intermediate heat exchanger using corrugated tubes. Int. J. Heat Mass Transf..

[10] Cruz G.G., Mendes M.A., Pereira J.M., Santos H., Nikulin A., Moita A.S. (2021). Experimental and numerical characterization of single-phase pressure drop and heat transfer enhancement in helical corrugated tubes. Int. J. Heat Mass Transf..

[11] Vicente P.G., Garcı A., Viedma A. (2004). Mixed convection heat transfer and isothermal pressure drop in corrugated tubes for laminar and transiton flow. Int. Commun. Heat Mass Transf..

[12] Zdaniuk G.J., Chamra L.M., Mago P.J. (2008). Experimental determination of heat transfer and friction in helically-finned tubes. Exp. Therm. Fluid Sci..

[13] Wang Y.-H., Zhang J.-L., Ma Z.-X. (2017). Experimental determination of single-phase pressure drop and heat transfer in a horizontal internal helically-finned tube. Int. J. Heat Mass Transf..

[14] Liu Z., Yue Y., She L., Fan G. (2019). Numerical analysis of turbulent flow and heat transfer in internally finned tubes. Front. Energy Res..

[15] Celen A., Dalkilic A.S., Wongwises S. (2013). Experimental analysis of the single phase pressure drop characteristics of smooth and microfin tubes. Int. Commun. Heat Mass Transf..

[16] Wang X., Ho J., Leong K., Wong T. (2018). Condensation heat transfer and pressure drop characteristics of R-134a in horizontal smooth tubes and enhanced tubes fabricated by selective laser melting. Int. J. Heat Mass Transf..

[17] Huang S., Wan Z., Tang Y. (2019). Manufacturing and single-phase thermal performance of an arc-shaped inner finned tube for heat exchanger. Appl. Therm. Eng..

[18] Chen H., Wan Z., Mo H., Huang S., Tang Y. (2019). Experimental studies on the compound thermo-hydraulic characteristics in a 3D inner finned tube with porous copper fiber inserts. Int. Commun. Heat Mass Transf..

[19] Huang S., Chen H., Zhang X., Wan Z., Tang Y. (2019). Experimental evaluation of thermal performance in a circular tube with Y-branch insert. Int. Commun. Heat Mass Transf..

[20] Tan X.-H., Zhu D.-S., Zhou G.-Y., Zeng L.-D. (2012). Experimental and numerical study of convective heat transfer and fluid flow in twisted oval tubes. Int. J. Heat Mass Transf..

[21] Wan Z., Tang Y. (2011). Characteristics of uncurled and reversely curled chip during orthogonal cutting. Int. J. Mach. Tools Manuf..

[22] Webb R. (1981). Performance evaluation criteria for use of enhanced heat transfer surfaces in heat exchanger design. Int. J. Heat Mass Transf..

[23] Kamboj K., Singh G., Sharma R., Panchal D., Hira J. (2017). Heat transfer augmentation in double pipe heat exchanger using mechanical turbulators. Heat Mass Transf..

[24] Sheikholeslami M., Gorji-Bandpy M., Ganji D. (2016). Effect of discontinuous helical turbulators on heat transfer characteristics of double pipe water to air heat exchanger. Energy Convers. Manag..

[25] Tang X., Dai X., Zhu D. (2015). Experimental and numerical investigation of convective heat transfer and fluid flow in twisted spiral tube. Int. J. Heat Mass Transf..

[26] Guo J., Fan A., Zhang X., Liu W. (2011). A numerical study on heat transfer and friction factor characteristics of laminar flow in a circular tube fitted with center-cleared twisted tape. Int. J. Therm. Sci..

[27] You Y., Fan A., Liu W., Huang S. (2012). Thermo-hydraulic characteristics of laminar flow in an enhanced tube with conical strip inserts. Int. J. Therm. Sci..

[28] Launder B.E., Spalding D.B. (1974). The numerical computation of turbulent flows. Comput. Methods Appl. Mech. Eng..

[29] Tu W., Wang Y., Tang Y. (2016). A numerical study on thermal-hydraulic characteristics of turbulent flow through a circular tube fitted with pipe inserts. Appl. Therm. Eng..

[30] Huang S., Wan Z., Wang Q., Tang Y., Yang X. (2017). Thermo-hydraulic characteristics of laminar flow in a circular tube with porous metal cylinder inserts. Appl. Therm. Eng..

[31] Li X.W., Meng J.A., Li Z.X. (2008). Experimental study of single-phase pressure drop and heat transfer in a micro-fin tube. Experi-Ment. Therm. Fluid Sci..

[32] Dizaji H.S., Jafarmadar S., Mobadersani F. (2015). Experimental studies on heat transfer and pressure drop characteristics for new arrangements of corrugated tubes in a double pipe heat exchanger. Int. J. Therm. Sci..

[33] Vicente P.G., Garcí A.A., Viedma A. (2002). Heat transfer and pressure drop for low Reynolds turbulent flow in helically dimpled tubes. Int. J. Heat Mass Transf..

